# Glucose, Cyc8p and Tup1p regulate biofilm formation and dispersal in wild *Saccharomyces cerevisiae*

**DOI:** 10.1038/s41522-020-0118-1

**Published:** 2020-02-13

**Authors:** Phu Van Nguyen, Vítězslav Plocek, Libuše Váchová, Zdena Palková

**Affiliations:** 1 Faculty of Science, Charles University, BIOCEV, Prague, Czech Republic; 2 Institute of Microbiology of the Czech Academy of Sciences, BIOCEV, Prague, Czech Republic

**Keywords:** Biofilms, Microbial genetics

## Abstract

*Saccharomyces cerevisiae* is a mainly beneficial yeast, widely used in the food industry. However, there is growing evidence of its potential pathogenicity, leading to fungemia and invasive infections. The medical impact of yeast pathogens depends on formation of biofilms: multicellular structures, protected from the environment. Cell adhesion is a prerequisite of biofilm formation. We investigated the adherence of wild and genetically modified *S. cerevisiae* strains, formation of solid–liquid interface biofilms and associated regulation. Planktonic and static cells of wild strain BRF adhered and formed biofilms in glucose-free medium. Tup1p and Cyc8p were key positive and negative regulators, respectively. Glucose caused increased Cyc8p levels and blocked cell adhesion. Even low glucose levels, comparable with levels in the blood, allowed biofilm dispersal and release of planktonic cells. Cyc8p could thus modulate cell adhesion in different niches, dependently on environmental glucose level, e.g., high-glucose blood versus low-glucose tissues in host organisms.

## Introduction

Fungal infections have become an increasing problem due to high associated mortality in immunosuppressed patients and limited availability of effective drug treatment, including an absence of biofilm-specific drugs. *Candida* and *Cryptococcu*s spp. are major human opportunistic yeast pathogens. However, *Saccharomyces cerevisiae* (a close relative of *C. glabrata*) that is widely used in the food industry, has begun to be considered as an opportunistic pathogen in recent years, having been implicated in a variety of infections ranging from vaginitis and cutaneous infections, to systemic bloodstream and organ infections in immunocompromised patients.^1,2^ *Saccharomyces* invasive infections are often clinically similar to invasive candidiasis.^3^ Clinical *S. cerevisiae* strains (and strains of non-clinical origin with virulence features) are distinct from laboratory strains. They are often resistant to factors such as oxidative stress, copper and high temperature^4,5^ and they can better survive in blood infection models. Clinical isolates often retain the ability to perform dimorphic switching between yeast form and pseudohyphae.^6^ As *S. cerevisiae* usually has a low susceptibility to amphotericin B and azoles,^7^ it can occupy niches, cleared of *C. albicans* and other yeasts in azole-treated patients.

Wild *S. cerevisiae* and *Candida* spp. adhere to different biotic and abiotic surfaces.^8,9^ Adhesion efficiencies depend on surface properties of adherent cells, such as the presence of specific proteins, adhesins, mediating cell-cell or cell-biotic/abiotic surface interactions. Adhesion is a key step allowing cells to occupy new niches in the host, and to establish multi-layered biofilm structures, providing yeast cells with multiple protection^10,11^ against the immune system and drug treatment. Hence, cell adhesion is an important factor in yeast virulence.^8^ For example, *C. albicans* cells adhered to an abiotic dental prosthetic were significantly more resistant to a range of antifungals than planktonic cells.^12^ Adhesins, mediating yeast cell adhesion to biotic (e.g., host tissues during infections) and/or abiotic (e.g., plastic) surfaces, include Epa adhesins of *C. glabrata*, Als adhesins of *C. albicans* and Flo11p adhesin of *S. cerevisiae*.^13–16^ Flo11p is also involved in other processes, including invasive growth and formation of complex structure of colony biofilms.^9,17–19^ Flo11p production is controlled by numerous factors that operate at different levels of Flo11p expression and function.^20^ Cyc8p (Ssn6p) and Tup1p are conserved factors regulating numerous processes, mostly as a co-repressor complex.^21^ In addition, Cyc8p and Tup1p antagonistically regulate Flo11p level and complexity of colony biofilms. Cyc8p represses the *FLO11* gene, preventing the formation of colony biofilms, whereas Tup1p antagonizes Cyc8p-mediated *FLO11* repression and, in addition, stabilizes the Flo11p protein by preventing its degradation.^22^

Here we show that Cyc8p negatively and Tup1p positively control adhesion of *S. cerevisiae* strains to plastic surfaces and subsequent formation of structured solid–liquid interface biofilm. The regulators influence adhesion of both shaken planktonic and static (sedimented) cells at any growth phase. In contrast to the wild strain, which is adhesive only in the absence of glucose, decreased level of Cyc8p also stimulates cell adhesion at high glucose concentrations. Glucose modulates cell adhesion and allows the release of planktonic cells from biofilms.

## Results

### Adhesion to plastic varies in different *S. cerevisiae* strains

**Fig. 1:**
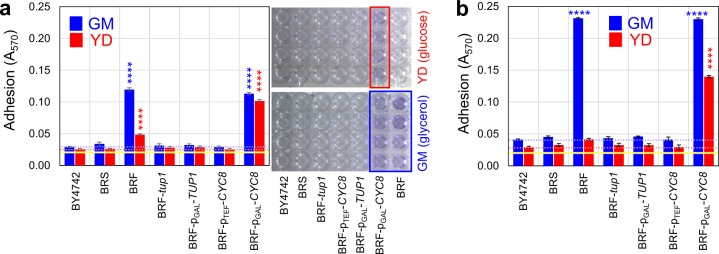
Adhesion to plastic of wild, domesticated and laboratory strains and strains with changed level of Tup1p and Cyc8p regulators. Adhesion of 24-h old static (**a**) or 18-h old shaken (**b**) cultures in GM (glycerol) in blue or YD (glucose) in red. Picture of plastic microtiter plate used for **a** measurements is shown. Blue and red dotted lines indicate A_570_ value measured with non-adhesive laboratory strain BY4742. Yellow line indicates background absorbance (BA). Four distinct experimental replicates (*n* = 4) were measured for each strain and condition with results expressed as the means and s.d.’s. The statistical significance of the variation relative to the non-adherent BY4742 was determined using an unpaired two-tailed *t*-test and GraphPad Prism6 software; ****p-value < 0.0001.

We first examined adhesion (the first step in biofilm formation) of non-isogenic strains BY4742 (a derivative of laboratory strain S288c), BRF (a wild strain) and domesticated strain BRS (Table 1), to polystyrene wells of microtiter plates. Two adhesion assays were performed. In the first assay, cells were inoculated directly into wells of microtiter plates and grown for 44 h (henceforth referred to as “static” cells) and then adherent cells were stained using crystal violet dye and quantified (Fig. 1a). In this assay, structured biofilm can develop (see below). In the second assay (Fig. 1b), planktonic cells were grown for 18 h in liquid medium with vigorous shaking (henceforth referred to as “planktonic” cells) and, after dilution to *A*_600_ = 1, were transferred to microtiter plate wells. After 3 h, adherent cells were stained as above and quantified. In this assay only the initial phase of biofilm formation, cell adhesion, was investigated. In both assays, strains were grown either in complete respiratory medium (GM) or in fermentative glucose medium (YD) (Fig. 1).

**Table 1:** Yeast strains.

Strains	Genotypes	References
BRF	*MATa*/*MATα*, wild strain isolate	^34^
BRF-Flo11p-GFP	*MATa*/*MATα, FLO11-GFP/FLO11*	^41^
BRS	*MATa*/*MATα*	^34^
BY4742	*MATα, his3Δ, leu2Δ, lys2Δ, ura3Δ*	Euroscarf.de
BRF-p_TEF_-*CYC8*	*MATa*/*MATα, FLO11-GFP/FLO11, nat1-*p_*TEF1*_*-CYC8/CYC8*	^22^
BRF-p_GAL_-*CYC8*	*MATa*/*MATα, FLO11-GFP/FLO11, cyc8∆::KanMX, nat1-*p_*GAL1*_*-CYC8*	^22^
BRF-p_GAL_-*TUP1*	*MATa*/*MATα, FLO11-GFP/FLO11, tup1∆::KanMX, nat1-*p_*GAL1*_*-TUP1*	^22^
BRF-p_CUP_-*CYC8*	*MATa*/*MATα, FLO11-GFP/FLO11, cyc8∆::KanMX, nat1-*p_*CUP1*_*-CYC8*	This study
BRF-p_CUP_-*TUP1*	*MATa*/*MATα, FLO11-GFP/FLO11, tup1∆::KanMX, nat1-*p_*CUP1*_*-TUP1*	This study
BRF-Cyc8p-GFP	*MATa/MATα CYC8-EGFP-kanMX/CYC8*	This study
BRF-Tup1p-GFP	*MATa/MATα TUP1-EGFP-kanMX/TUP1*	This study
BRF-p_GAL_-*CYC8-*p_CUP_-*TUP1*	*MATa/MATα, tup1∆::loxP, cyc8∆::loxP, KanMX-p*_*CUP1*_*-TUP1, nat1-*p_*GAL1*_*-CYC8*	^22^
BRF-p_GAL_-*TUP1-*p_CUP_-*CYC8*	*MATa/MATα, tup1∆::loxP, cyc8∆::loxP, nat1-*p_*GAL1*_*-TUP1, KanMX-*p_*CUP1*_*-CYC8*	^22^

Only wild strain BRF was significantly adhesive. Neither BY4742 nor the BRS strain adhered to the plastic surface in any of the conditions tested (Fig. 1). Adhesion efficiency of both static and planktonic BRF cells was much higher in glucose-free GM than in glucose-rich YD. Moderate adhesion of BRF static cells in YD (Fig. 1a) could be due to glucose consumption and thus its decreased level during prolonged cultivation. Adhesion of planktonic GM-pre-grown BRF cells was on average twice as high as that of static cells (compare Fig. 1a, b).

### Cyc8p and Tup1p conversely regulate cell adhesion

Next, we asked whether Cyc8p and Tup1p, which regulate formation of structured colony biofilms^22^ are also involved in BRF cell adhesion to plastic. We therefore assayed the adhesion capability of BRF-derived strains with modified levels of Cyc8p and Tup1p (Table 1). Deletion of gene *CYC8* is lethal in the BRF strain, therefore we used strain BRF with one *CYC8* allele deleted and the second allele placed under the control of the p_GAL_ inducible promoter (BRF-p_GAL_-*CYC8*) generating only a low basal level of *CYC8* expression in the absence of galactose. Similarly, in order to control the level of *TUP1* expression, we used strain BRF-p_GAL_-*TUP1* with one *TUP1* allele deleted and the second controlled by p_GAL_. As in the case of BRF-p_GAL_-*CYC8*, the BRF-p_GAL_-*TUP1* strain expressed only a low basal level of *TUP1* in the absence of galactose. We also used a strain, deleted in both *TUP1* alleles (BRF-*tup1*). Loss of expression or low basal expression of *TUP1*, or constitutive over-expression of *CYC8*, completely eliminated the adhesion capability of the BRF strain (Fig. 1; strains BRF-*tup1*, BRF-p_GAL_-*TUP1*, and BRF-p_TEF_-*CYC8*). In contrast, a decreased level of *CYC8* did not change adhesion capability of BRF in GM but greatly increased cell adhesion of both planktonic and static cells in high-glucose YD (Fig. 1; strain BRF-p_GAL_-*CYC8*).

**Fig. 2:**
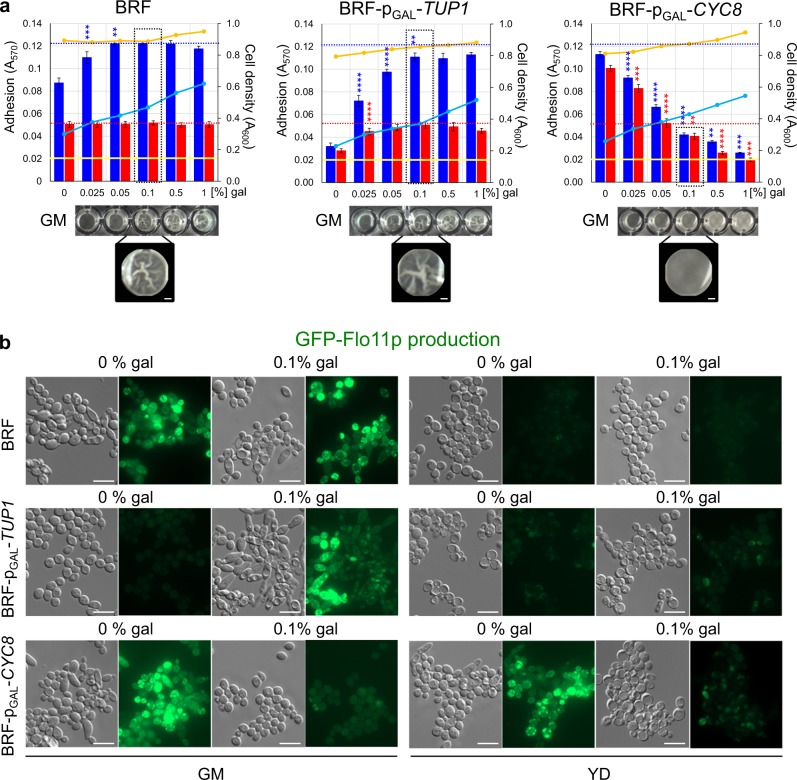
Effect of galactose on adhesivity, biofilm formation and Flo11p-GFP production of static BRF, BRF-p_GAL_-*TUP1* and BRF-p_GAL_-*CYC8* cells in GM and YD. **a** Adhesivity of 24-h old static cultures in GM (blue bars) or YD (red bars) treated with 0–1% galactose (gal) was analyzed. Biomass yield (*A*_600_) in GM (blue curve) and in YD (orange curve). Blue and red dotted lines indicate maximal *A*_570_ value measured with BRF. Yellow line indicates BA. Solid-liquid interface biofilm formed by BRF and galactose-induced BRF-p_GAL_-*TUP1* in GM is shown at the bottom; bar, 1 mm. Four distinct experimental replicates (*n* = 4) were measured for each strain and condition with results expressed as the means and s.d.’s. The statistical significance of the variation between two succeeding galactose concentrations was determined using an unpaired two-tailed *t*-test and GraphPad Prism6 software; ****p-value < 0.0001, ***p-value < 0.001, and **p-value < 0.01. **b** Cell morphology (differential interference contrast, DIC) and presence of Flo11p-GFP in cells of respective strains grown in GM or YD and induced/non-induced by 0.1% galactose. Bar, 10 μm.

Expression of either *TUP1* or *CYC8* is inducible by galactose in BRF-p_GAL_-*TUP1* and BRF*-*p_GAL_*-CYC8*, respectively. Galactose, like glucose, is a fermentative sugar that increases growth rate, and could reduce cell adhesion, as it does other biofilm properties,^23^ at high concentration. Therefore, we first estimated biomass yield and cell adhesion after 24 h cultivation in GM or YD with different galactose concentrations. As expected, increased BRF biomass yield in static cultivation correlated with galactose concentration in GM (Fig. 2a). BRF adhesion in GM increased slightly up to 0.05% galactose, possibly because of incomplete well surface coverage by cells in poor GM. Cells in 0.05–0.5% galactose exhibited uniform adhesion, independently of enhanced growth. Even the highest initial galactose concentration (1%) did not significantly decrease cell adhesion and so structured biofilm was formed (Fig. 2a). As expected, galactose did not influence BRF growth and adhesion in YD medium.

BRF-p_GAL_-*TUP1* and BRF*-*p_GAL_*-CYC8* biomass accrual in GM with galactose was only slightly lower than that of BRF, but adhesion of these two strains varied greatly, depending to galactose concentration: due to the induction of *TUP1* or *CYC8* expression (Fig. 2a). BRF-p_GAL_-*TUP1* adhesion increased in GM as galactose concentration ranged from 0 to 0.1% and was constant between 0.1 and 1% galactose. Wrinkled biofilms, visible to the naked eye, were formed over a range of 0.05–1% galactose. BRF*-*p_GAL_*-CYC8* adhesion decreased over the entire concentration range (0.025–1% galactose). In contrast to GM, the presence of galactose in YD did not affect BRF background adhesion and only moderately increased adhesion of BRF-p_GAL_-*TUP1*. On the other hand, adhesion of BRF*-*p_GAL_*-CYC8* (non-induced) in YD was as high as in GM and was diminished by galactose induction of *CYC8* with the same concentration-dependent profile in both media. The p_GAL_ promoter is inducible by galactose but also repressible by glucose, which together could result in a merely moderate increase in p_GAL_-driven expression at high (2%) glucose concentration.^24^ Therefore, we also assayed the effect of *TUP1* and *CYC8* induction on cell adhesion in GM and YD, using strains BRF-p_CUP_-*TUP1* and BRF*-*p_CUP_*-CYC8*, inducible by copper. The results (Supplementary Fig. 1) were comparable to those obtained by galactose induction, demonstrating that galactose induction is not deficient in the presence of glucose. To minimize the effect of cell accrual, 0.1% galactose was chosen for further experiments, producing uniform adhesion of BRF, maximal adhesion of BRF-p_GAL_-*TUP1* and a drop in BRF*-*p_GAL_*-CYC8* adhesion to below the BRF-adhesion threshold.

Next, we used strains BRF-p_GAL_-*TUP1*-p_CUP_-*CYC8* and BRF-p_GAL_-*CYC8*-p_CUP_-*TUP1* (Table 1), in which both *CYC8* and *TUP1* were controlled by inducible promoters to assay the balancing effect of Cyc8p and Tup1p levels on cell adhesion in 24-h-old static cultures in GM and YD (Supplementary Fig. 2). In GM medium, induction of *CYC8* and *TUP1* (using galactose plus Cu^2+^), or of *TUP1* (by galactose or Cu^2+^) in the absence of Cyc8p, lead to adhesion comparable with BRF adhesion. In contrast, induction of *CYC8* (by galactose or Cu^2+^) in the absence of Tup1p eliminated adhesion almost completely. Adhesion of both strains without induction of either regulator (no galactose, no Cu^2+^) was also strongly reduced, but was still higher than in the presence of high Cyc8p. In YD medium, low adhesion, comparable with BRF, was found when strains were induced by galactose and Cu^2+^ (high Tup1p and Cyc8p) as well as in the absence of their induction (low Tup1p and Cyc8p). Adhesion was almost completely eliminated when Cyc8p was high and Tup1p low (galactose or Cu^2+^ induction of only *CYC8*). When Cyc8p was low and Tup1p high (galactose or Cu^2+^ induction of only *TUP1*), adhesion increased as in the case of non-induced strain BRF-p_GAL_-*CYC8*, in which *TUP1* is controlled by its native promoter (compare Supplementary Fig. 2 and Fig. 1a). Hence, increased adhesion in YD due to the absence of *CYC8* also requires the presence of Tup1p.

We further determined the correlation between strain adhesion and Flo11p expression. Cells of the BRF, BRF-p_GAL_-*TUP1* and BRF*-*p_GAL_*-CYC8* strains grown in GM or YD with 0 or 0.1% galactose in the wells were collected to estimate Flo11p-GFP presence (Fig. 2b). Flo11p-GFP level strictly correlated with strain adhesion ability, being high in BRF in GM (with or without galactose), BRF-p_GAL_-*TUP1* in GM with galactose (induced *TUP1* expression) and BRF*-*p_GAL_*-CYC8* in either GM or YD without galactose (diminished *CYC8* expression). In all other conditions, Flo11p-GFP fluorescence was negligible.

These data showed that Cyc8p and Tup1p conversely regulate cell adhesion involving the Flo11p adhesin, which is important in abiotic surface adhesion. Moreover, as with cell adhesion, Flo11p expression is strongly de-repressed in high glucose when Cyc8p level drops (YD without galactose) and Tup1p is present.

### Cyc8p and Tup1p conversely regulate formation of structured biofilm

**Fig. 3:**
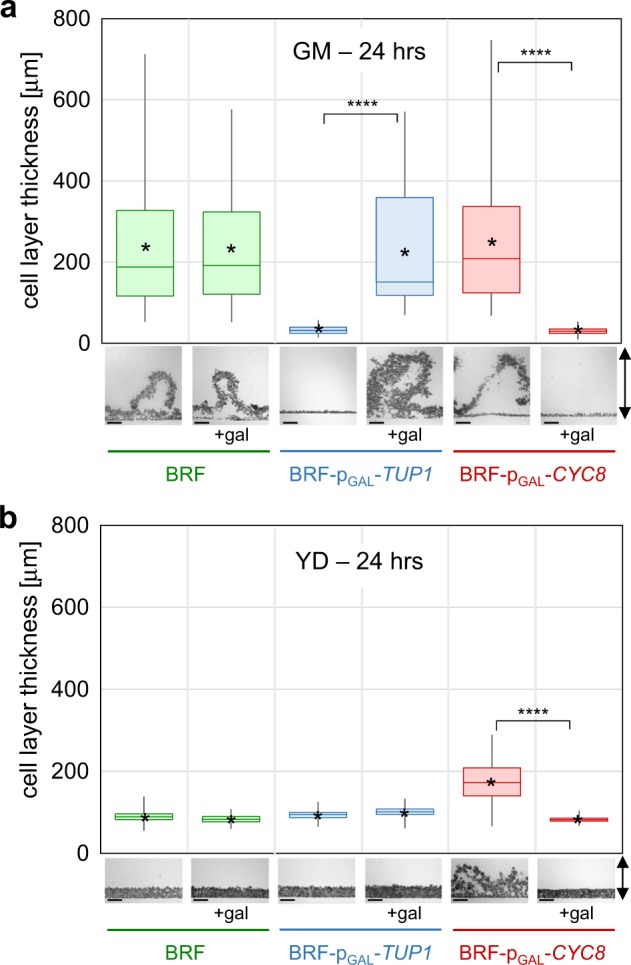
Thickness of biofilms and non-adherent cell layers formed by BRF, BRF-p_GAL_-*TUP1* and BRF-p_GAL_-*CYC8* cells. Vertical cross-sections of 24-h-old static cultures in GM (**a**) or YD (**b**) without galactose or treated with 0.1% galactose (gal) were used for measurement of thickness of biofilms and non-adherent cell layers. The box plots show distribution of thickness measured at different positions (deep and shallow wrinkles) within biofilms and non-adherent cells (box extends from the 25th to 75th percentiles with center line representing the median and whiskers from Min to Max, asterisk indicates the mean). >200 different positions from 3 to 5 distinct cross-sections were measured for each plot using ImageJ. The statistical significance of the variation between galactose-treated and untreated samples was determined using an unpaired two-tailed *t*-test and GraphPad Prism6 software; ****p-value < 0.0001. Pictures at bottom show examples of cross-sections used for the measurement; black arrows indicate vertical direction of cross-sections. Bar, 100 μm.

Next, we determined whether differences in adhesion mediated by Cyc8p and Tup1p, were reflected in formation of wrinkled solid–liquid interface biofilms. BRF, BRF-p_GAL_-*TUP1* and BRF-p_GAL_-*CYC8* strains were grown for 24 and 48 h in GM and YD with and without galactose and the minimum and maximum thickness of structured biofilm wrinkles and of non-adherent cell layers, respectively, were measured. In accordance with cell adhesion results, the formation of the three-dimensional structure of the wrinkled biofilm was also dependent on the presence of Tup1p and was inhibited by increased Cyc8p expression (Fig. 3). The thickness of 24-h-old biofilm formed by BRF, BRF-p_GAL_-*TUP1* (induced by galactose) and BRF-p_GAL_-*CYC8* (non-induced) was greater in GM medium (Fig. 3a) than that of biofilm formed by BRF-p_GAL_-*CYC8* (non-induced) in YD (Fig. 3b). This indicates that low Cyc8p is sufficient to induce efficient adhesion and initiation of biofilm formation in the presence of glucose, while the development of fully structured biofilm probably requires other factors that are absent in high glucose conditions. Further cultivation did not significantly influence biofilm thickness as 48 h-old biofilms exhibited a thickness, similar to that of 24 h-old biofilms (compare Fig. 3 and Supplementary Fig. 3).

### Glucose disrupts biofilm and releases adhered cells

**Fig. 4:**
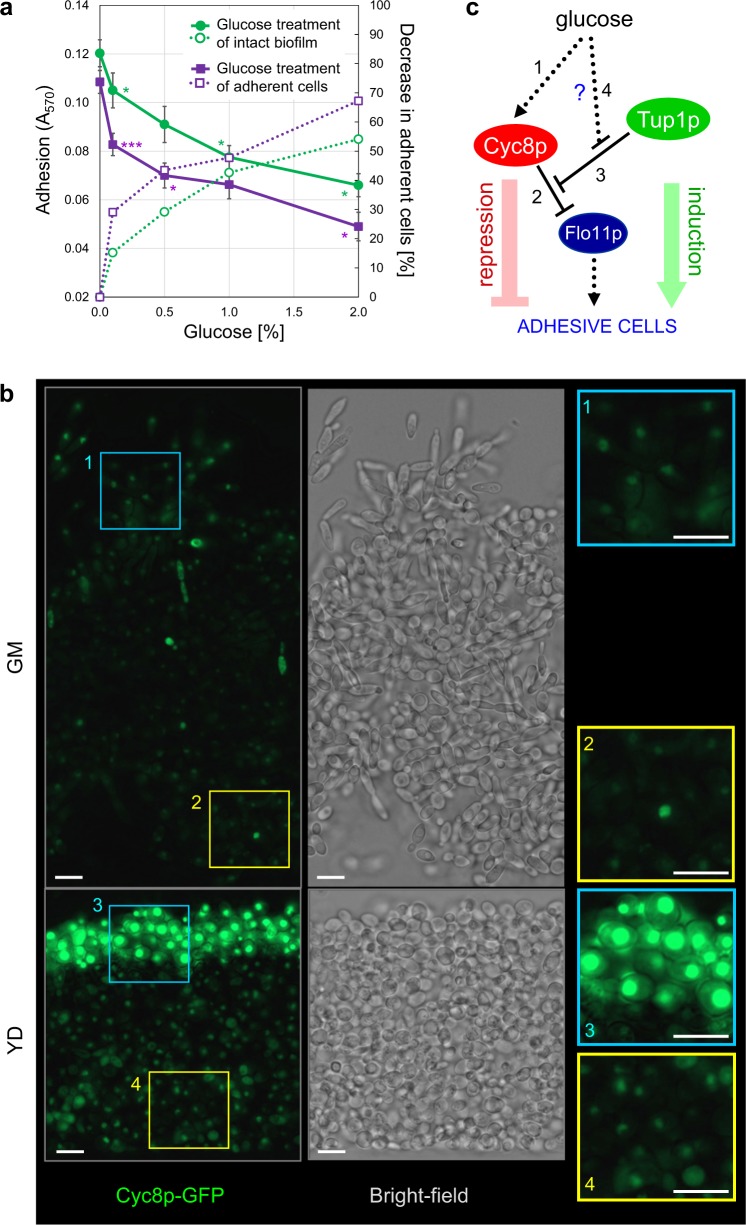
Role of glucose in Cyc8p and Tup1p regulation of yeast adhesion and biofilm formation: experimental data and scheme. **a** Glucose was added to the intact biofilm in GM (green) or to the adherent cells after medium removal (violet). Solid lines, cell adhesion after glucose treatment; dotted lines, decrease in percentage of adhesive cells due to glucose treatment. Experiments were conducted in quadruplicate (distinct samples, *n* = 4) with results expressed as the means and s.d.’s. The statistical significance of the variation between two succeeding galactose concentrations was determined using an unpaired two-tailed t test and GraphPad Prism6 software; ***p-value < 0.001 and *p-value < 0.05. **b** Vertical cross-sections of 24-h-old static cultures of BRF-Cyc8p-GFP strain in GM or YD; Cyc8p-GFP fluorescence in green, cells are visible in bright-field. Cells in Insets “1–4” are shown at higher magnification, indicating distribution and intensity of fluorescence of Cyc8p-GFP in different biofilm/non-adherent cell regions. Bar, 10 μm. A representative experiment of three (*n* = 3) independent experiments is shown. **c** Model of glucose function: Glucose induces Cyc8p expression/function “1”, subsequently Cyc8p represses *FLO11* expression “2” and cell adhesion is blocked. This effect would be enhanced if glucose also negatively affects Tup1p function “4”. In glucose absence, Cyc8p level is decreased and its function is inhibited via Tup1p “3” as described.^22^ Subsequently Flo11p is produced and contributes to cell adhesion. Arrow, induction; blunt line, repression. Dotted line, additional proteins may participate in the effect. Light-color blunt line (red) and arrow (green) indicate effects of the regulators on cell adhesion.

Intriguingly, decreased level of Cyc8p greatly increased cell adherence and biofilm formation in the presence of glucose (in YD). We therefore tested the hypothesis that level of glucose could modulate release of biofilm cells. Different concentrations of glucose were applied for 4 h either to intact biofilm of strain BRF (formed during 44 h static cultivation in GM) or to the same biofilm, but after gentle removal of liquid medium with non-adherent cells. In both cases, glucose caused significant biofilm disruption and release of adherent cells (Fig. 4a). Even a concentration of 0.1% glucose decreased adhesion by ~15% and ~30%, respectively. Two percent glucose, added after removal of the medium, caused release of almost 70% of adherent cells (Fig. 4a).

### Glucose increases amount of Cyc8p in cell nuclei

To further clarify the relationship between glucose and Cyc8p function, we constructed BRF strains with either Cyc8p or Tup1p tagged with GFP (strains BRF-Cyc8p-GFP and BRF-Tup1p-GFP, Table 1). Both Cyc8p-GFP and Tup1p-GFP localize to the nucleus. We then used these strains to estimate levels of Cyc8p and Tup1p in non-adherent static cells grown in the presence of glucose (YD) and in biofilms grown in the absence of glucose (GM). Biofilms and non-adherent cells were grown in microtiter plates for 24 h and vertical cross-sections analyzed by fluorescence and bright-field microscopy. The level of Cyc8p-GFP was significantly higher in non-adherent cell layers in YD, than in biofilms in GM (Fig. 4b). In addition, the Cyc8p-GFP level was high in nuclei of surface cell layers, in particular in YD, and lower in internal regions. This may be because the glucose had already been spent in lower cells that were not in direct contact with the medium. Hence, the major difference in Cyc8p level between biofilms and non-adherent cell layers concerns surface areas. In contrast to Cyc8p, no visible difference in Tup1p-GFP level was observed between biofilms and non-adherent cell layers (Supplementary Fig. 4).

### Tup1p and Cyc8p regulate adhesion of both planktonic and static cells independently of growth phase

Next we asked whether cell adhesion is regulated by Tup1p and Cyc8p identically or differently, depending on the cell state, such as different cell growth phases (exponential, diauxic, and stationary phase) or different lifestyle (static versus planktonic cells). We also tested the speed of the cellular response (cell adhesion or release) to increased Tup1p or Cyc8p levels by treating cells with inducer (galactose) for 6 or 10 h.

**Fig. 5:**
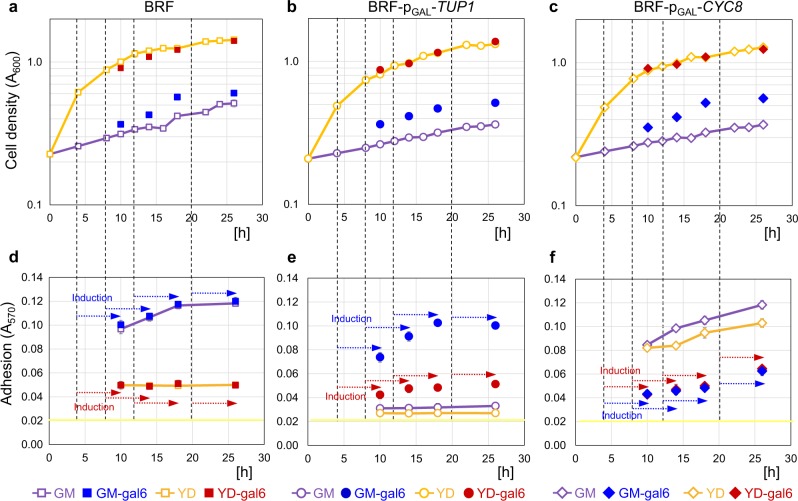
Effect of Tup1p or Cyc8p induction on adhesivity of cells in different growth phases of static cultures. **a**–**c** Strain growth curves in GM and YD. gal6, galactose was added 6 h before measurement of the biomass (*A*_600_). **d**–**f** Adhesivity of the strains without induction and after 6 h of galactose induction. Arrows (**d–f**) indicate interval of galactose presence in relation to time-points (indicated by dashed lines), in which galactose was added. Yellow line indicates BA. Experiments were conducted in quadruplicate (distinct samples, *n* = 4) with results expressed as the means and s.d.’s.

In the “static” setup, BRF-p_GAL_-*TUP1*, BRF*-*p_GAL_*-CYC8*, and BRF were grown in GM or YD in wells, *TUP1* or *CYC8* expression was induced by galactose at specific time-points for either 6 (Fig. 5d–f) or 10 h (Supplementary Fig. 5d–f) and adhesion analyzed. In parallel, we measured growth curves of all strains (Fig. 5a–c and Supplementary Fig. 5a–c). Adhesion of BRF in GM reached a similar level, independently of galactose presence and also of cell density, which is higher in galactose-treated cultures. The BRF adhesion maximum in GM was reached at 17 h at a cell density, corresponding to *A*_600_ ~ 0.4 and it remained constant later (Fig. 5d), despite continued growth of the strain (Fig. 5a). As expected, BRF-p_GAL_-*TUP1* with low *TUP1* expression exhibited only basal adhesion in GM. Induction of *TUP1* expression by 6-h-galactose treatment at any time-point was not sufficient to induce the maximal level adhesion reached by BRF (compare Fig. 5d, e). As with BRF, adhesion of younger BRF-p_GAL_-*TUP1* populations was slightly lower than that of older ones (Fig. 5e). Adhesion, similar to that of BRF, was reached only after 10-h induction of *TUP1* expression (Supplementary Fig. 5e). Adhesion of BRF*-*p_GAL_*-CYC8* with low *CYC8* expression in GM (Fig. 5f and Supplementary Fig. 5f) resembled that of BRF (Fig. 5d and Supplementary Fig. 5d). Induction of *CYC8* expression by galactose diminished BRF*-*p_GAL_*-CYC8* adhesion almost independently of treatment period (Fig. 5f and Supplementary Fig. 5f). As expected, in YD, BRF exhibited only basal adhesion, independently of galactose treatment (Fig. 5d and Supplementary Fig. 5d). A low level of Tup1p in BRF-p_GAL_-*TUP1* caused a slight decrease in this basal adhesion in YD, which reverted back after galactose induction of *TUP1* (Fig. 5e and Supplementary Fig. 5e). A low level of Cyc8p always increased adhesion efficiency in YD almost to the same level as in GM and galactose induction of *CYC8* diminished adhesion similarly in YD and GM (Fig. 5f and Supplementary Fig. 5f).

**Fig. 6:**
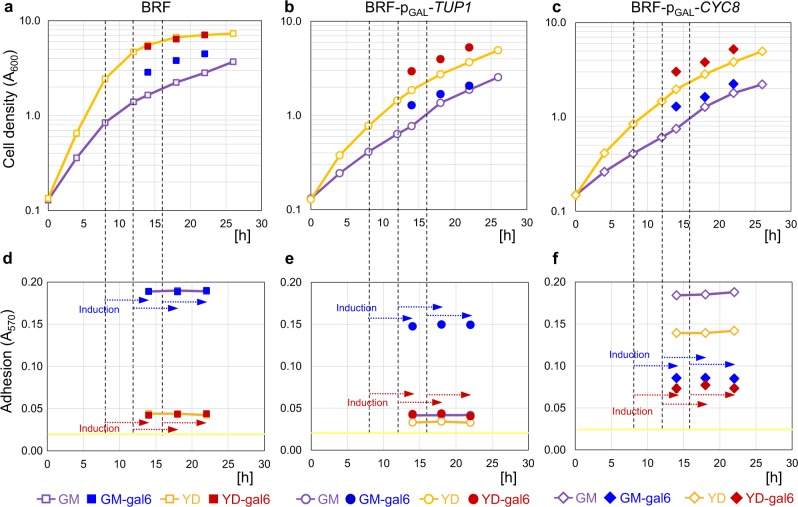
Effect of Tup1p or Cyc8p induction on adhesivity of planktonic cells from different growth phases of shaken cultures. **a**–**c** Strain growth curves in GM and YD. gal6, galactose was added 6 h before measurement of the biomass (*A*_600_). **d**–**f** Adhesivity of the strains without induction and after 6 h of galactose induction. Arrows (**d**–**f**) indicate period of galactose treatment in relation to time-points (indicated by dashed lines) in which galactose was added. Yellow line indicates BA. Experiments were conducted in quadruplicate (distinct samples, *n* = 4) with results expressed as the means and s.d.’s.

In the “planktonic” setup (Fig. 6 and Supplementary Fig. 6), the same number of planktonic cells was applied to the wells in each time-point and thus the adhesion efficiency was not influenced by cell numbers in wells. In contrast to static cultures, adhesion efficiency was completely independent of cell growth phase and reached highest levels under conditions, permitting adhesion (Fig. 6d–f and Supplementary Fig. 6d–f). Adhesion of planktonic BRF cells was independent of galactose presence (similarly to static cultures), being high in GM and basal in YD (Fig. 6d and Supplementary Fig. 6d). BRF-p_GAL_-*TUP1* adhesion was basal in GM and YD and increased only in GM due to galactose-mediated *TUP1* induction, reaching higher values after 10 than 6 h of galactose treatment (Fig. 6e and Supplementary Fig. 6e). BRF*-*p_GAL_*-CYC8* adhesion was comparable with BRF in GM but, again, significantly higher in YD (Fig. 6f and Supplementary Fig. 6f). In contrast to static cells, 6-h-galactose induction of *CYC8* led to a smaller decrease in adhesiveness (Fig. 6f) than 10 h-treatment (Supplementary Fig. 6f) and neither reduced adhesion to BRF basal level (compare Fig. 6d, f and Supplementary Fig. 6f, d).

In summary, these data indicate that Cyc8p and Tup1p mediated regulation is robust, functioning similarly in static and planktonic cells and being only moderately influenced by cell culture growth phase. In both static and planktonic cultivation, the effect of Cyc8p induction is similar after 6 and 10 h and is thus quicker than that of Tup1p which is higher after 10 than after 6 h. However, the decrease in adhesion, compared with BRF, is higher in static than in planktonic cells upon Cyc8p induction. These observations are consistent with other data indicating that an increase in Cyc8p level causes release of adherent cells from already formed biofilms.

## Discussion

We have shown that adhesion of wild strain BRF to an abiotic solid surface, the related Flo11p expression, and the formation of structured biofilm at a solid–liquid interface, are regulated conversely by Tup1p and Cyc8p. Furthermore, we have revealed that Tup1p-induced adhesion is efficient in glucose-free conditions, whereas Tup1p presence is necessary but not sufficient to induce adhesion of cells grown in the presence of glucose. In contrast, Cyc8p-mediated repression of adhesion is efficient and comparable in both conditions, thus demonstrating that Cyc8p is an important player in glucose-regulated cell adhesion and biofilm formation.

Adhesion of both static and planktonic cells is regulated by Cyc8p and Tup1p, with only slight differences. Relatively homogeneous planktonic cells adhered independently of growth phase and with higher efficiency than their static counterparts. Tup1p-mediated adhesion efficiency slightly increased in older static cultivations. This indicates that more adhesive and less adhesive cells are present near to the plastic surface in static cultivations and that the frequency of more adhesive cells slightly increases as the biofilm develops. Static biofilm cells also respond faster than planktonic cells to an increase in Cyc8p by massive release from the plastic surface. Cell response to *TUP1* induction was slower, but still quicker in static than in planktonic cells.

BRF cell adhesion and biofilm formation is strictly dependent on medium composition, being high in the absence of glucose (“permissive”) and low in high glucose (“non-permissive”) conditions. Tup1p is a key activator of cell adhesion under permissive conditions, but even increased Tup1p levels had negligible effect under non-permissive conditions when Cyc8p was expressed from its native promoter. In contrast, increased Cyc8p level seems to exert a strong effect under non-permissive conditions, counteracting the Tup1p and Flo11p functions, potentially via mechanisms, described recently.^22^ Glucose may thus regulate Cyc8p level and/or repressor activity (Fig. 4c). Several factors participating in glucose regulation, such as Nrg1p, Mig1p, and Sfl1p, were shown to interact with, and influence the function of, the Cyc8p-Tup1p complex.^11,25,26^ However, neither of these factors is involved in antagonistic Cyc8p and Tup1p regulations.^22^ Little information is available on environmental regulation of the *CYC8* gene at the transcription level, but genome-wide transcriptomic screens showed the level of *CYC8* mRNA to be ~2.4 times higher after 15 min treatment in 2% glucose than in 0.05% glucose^27^ and *CYC8* mRNA level gradually decreased during prolonged cultivation in glucose YPD medium,^28^ possibly due to glucose consumption. Here we show that the level of Cyc8p-GFP is much higher in nuclei of cells at surface layers of non-adherent cells grown in glucose-rich YD medium than in nuclei of cells in biofilms grown in glucose-free GM. Altogether, these data support the hypothesis that Cyc8p level itself is regulated by glucose (Fig. 4c), though the existence of a specific, as yet unidentified, factor influencing repressive Cyc8p function, cannot be excluded. Cyc8p level is affected by glucose mainly in surface cells, which are in contact with the fluid (medium). This is in agreement with the observed efficient release of solid–liquid interface biofilm upon the addition of glucose. In principle, in this way, whole biofilms and/or individual planktonic cells could be efficiently released from solid/semi-solid supports.

**Fig. 7:**
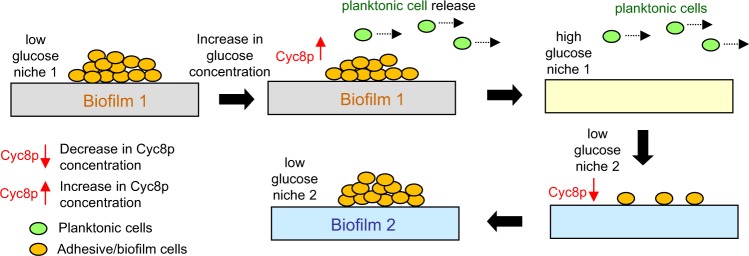
Model of role of glucose in formation of solid–liquid interface biofilm. Level of environmental glucose can regulate switch between cell adhesion/biofilm formation and release of planktonic cells in structured heterogeneous environment (organism). Biofilm 1, primary biofilm from which planktonic cells may be released when glucose concentration increases. Released cells may then settle in new low-glucose niches, in which they form secondary biofilm (Biofilm 2).

Involvement of a glucose-responsive factor in adhesion and biofilm formation could play an important role in yeast virulence and in systemic and biofilm infections (Fig. 7). When a yeast cell settles in a low-glucose niche (tissue), the level/function of Cyc8p is lowered, allowing Tup1p to mediate cell adhesion and biofilm development (Fig. 7, “Biofilm 1”). Contact of biofilms with higher glucose environments (such as blood/plasma) causes an increase in Cyc8p level/function and subsequent decrease in adhesion and release of free planktonic cells. These cells may then be dispersed until they reach another low glucose niche (another tissue), in which Cyc8p level/function is again repressed, allowing yeast to adhere and form biofilm (Fig. 7, “Biofilm 2”) therein. By this mechanism, planktonic cells and biofilms are able to spread and survive in a heterogeneous environment, such as a host organism. As even 0.1% (=~5.5 mM) glucose concentration is sufficient to release 15–30% of cells from the biofilm (Fig. 4a), our model is relevant under physiological conditions, since normal blood glucose concentration is below 7.8 mM (2 h post-prandial).^29^ Transient glycosuria in diabetics or some pregnant women may also cause yeast biofilm to spread to the genitourinary tract.

It has been shown that, in addition to anticancer treated immunocompromised patients, diabetic patients represent another group, at high-risk of invasive fungal infections^30^ and examples of *S. cerevisiae* infection have been reported in these patients.^1^ An increase in glucose level has been detected in blood and other body niches such as urine, intestinal fluid, mucus, sweat/perspiration and saliva^31^ and is a known risk-increasing factor in fungal infections for two main reasons: Increased glucose benefits yeast cell growth and decreases the effect of some antifungal drugs.^30,32^ Here we speculate about a third glucose-related risk—increase of glucose in some niches could lead to release of adherent cells and their dispersal within the organism, potentially followed by development of new biofilms as new outbreaks of infection. We show that Cyc8p is a key factor in this process and, together with Cyc8p and Tup1p mediated antiregulation, is effective in both adherent and planktonic cells and mostly independent of growth phase. We also proved that an increase in glucose concentration causes release of adherent cells from complex biofilm structures. Both Cyc8p and Tup1p are conserved among yeast and information concerning their function in *Candida* spp. cell adhesion is scarce. One indication of a positive role for Tup1p in adhesion of *C. albicans* comes from findings that a *tup1*Δ strain forms only pseudohyphae with reduced adhesion to keratinocytes.^33^ Hence, besides Cyc8p and Tup1p being potential risk factors and therapeutic targets in (so far) less frequent *S. cerevisiae* infections, Cyc8p and Tup1p orthologues may be involved in adhesion, biofilm formation and cell release in *Candida* spp., in particular *C. glabrata*, a close relative of *S. cerevisiae*.

## Methods

### Yeast strains and media

Strains used in this study (Table 1) were derived from wild strain BRF^18,34^ from the collection of the Institute of Chemistry, Slovak Academy of Sciences (collection number CCY 21-4-97). Strain BY4742 was obtained from Euroscarf (collection number Y10000). For adhesion assays, strains were grown in GM (3% glycerol, 1% yeast extract) or YD (2% glucose, 1% yeast extract) media without any additives, or supplemented with galactose and/or Cu^2+^ (CuSO_4_) in final concentration as described in the Result section.

### Strain constructions

*CYC8* and *TUP1* gene knock-outs were performed by transforming the cells with deletion cassettes generated by PCR from plasmid pUG6.^35^ Strains with C terminal GFP fusions were constructed using a GFP-KanMX integrative cassette, amplified by PCR from plasmid pKT127.^36^ Strains expressing *TUP1* or *CYC8* under the control of inducible promoter p_CUP_ were constructed by integration of p_*CUP1*_-natNT2 cassettes amplified from the pYM-N2 plasmid.^37^ Yeast cells were transformed using a standard lithium acetate/polyethylene glycol method.^38^ Positive transformants were selected on GMA (GM, 2% agar) supplemented with G418 (200 mg/l) or nourseothricin (100 mg/l). Correct genomic integration of cassettes was verified by PCR using specific primers and by sequencing. The primers and plasmids are listed in Supplementary Table 1.

### Cell cultivation for adhesion assays

Biofilm cultures (static cells). Cells from overnight cultures in GM or YD (28 °C, with shaking) were harvested. Biomass was inoculated into fresh GM or YD, respectively, to a concentration of 0.3 mg wet weight/ml). For biofilm cultivation, 150 μl of cell suspension was pipetted into each well of a polystyrene 96 well microtiter plate V400917 from GAMA group Inc., CZ (four independent replicates per strain and condition) and incubated at 28 °C. If needed, galactose and/or Cu^2+^ (CuSO_4_) was added to the required final concentration. Cell absorbance (*A*_600_) was determined at indicated time-points. In parallel, cell adhesion was determined.

Shaken cultures (planktonic cells): 10 ml of cell suspension was cultivated at 28 °C in an Erlenmeyer flask with vigorous shaking (150 rpm) and cell density determined as *A*_600_. If needed, galactose and/or Cu^2+^ (CuSO_4_) was added to the required final concentration. At appropriate time-points, cells were harvested, washed and resuspended in water to *A*_600_ = 1. Then 150 μl of the cell suspension (approximately 1–3 × 10^7^ cells/ml) was pipetted into the wells of a 96 well microtiter plate (four independent replicates per strain and condition) and incubated at 28 °C. Adhesion was determined after 3 h incubation.

### Adhesion assays

The adhesion assay^9,39^ was performed with modifications. The liquid (medium or water) was removed and the microtiter plates thoroughly washed by submerging three times in water. One hundred and fifty microliter of 1% crystal violet dye was added to each well and plates were incubated at room temperature with gentle shaking. After 15 min, the dye solution was removed and the plates were washed three times with distilled water. Afterwards, 150 µl of 95% ethanol was added to each well to elute the dye from attached cells. Hundred microliter of the crystal violet eluate from each well was transferred to a new microtiter plate and the absorbance (*A*_570_) measured by Epoch Microplate Spectrophotometer (Biotek). *A*_570_ value reflects the number of adherent cells and was taken as a measure of relative efficiency of adhesion. In control measurements, the complete procedure was performed in a microtiter plate without cells. The measured value, 0.02 was indicated in the graphs as the background absorbance (BA).

### Release of adherent cells by glucose

Glucose was added (i) directly to the well containing the intact biofilm in GM or (ii) to the biofilm after removal of GM with non-adhered cells and washing, to desired final concentration. After 4 h incubation, all non-adherent cells were removed by washing and adhesive cells quantified as above.

### Biofilm imaging and cell microscopy

Images of biofilms in wells of microtiter plate were captured in incident and/or transmitted light. A ProgRes® CT3 CMOS camera with a Navitar objective and NIS Elements software (Laboratory Imaging, s.r.o, Prague, CZ) were used. For thickness analyses of biofilms and non-adherent cell layers and analyses of Tup1p-GFP and Cyc8p-GFP expression, biofilms and non-adherent cell layers were fixed with 4% agarose and sectioned using a Leica VT1200S vibrating microtome.^40^ Re-suspended cells or sections of biofilms/non-adherent cell layers were observed using Carl Zeiss Axio Observer.Z1 fluorescence microscope equipped with Axiocam 506 and a C-Apochromat 10×/0.45 W objective (for thickness analyses) or a C-Apochromat 63×/1.20 W (for GFP expression) using ZEN 2012 (blue edition) software. Filter sets for GFP (excitation 450–490 nm; emission 500–550 nm), differential interference contrast (DIC) or bright field were used. The thickness of biofilms/non-adherent cell layers was measured from images using ImageJ (version 1.52a).

### Reporting summary

Further information on research design is available in the Nature Research Reporting Summary linked to this article.

## Supplementary information

Supplementary Information

Reporting Summary

## Data Availability

The data that support the findings of this study are included in the article, its supplementary information files, or are available from the corresponding author upon reasonable request.
