# Supplementary material for: Glucose, Cyc8p and Tup1p regulate biofilm formation and dispersal in wild Saccharomyces cerevisiae
Source: NPJ Biofilms Microbiomes. 2020 Feb 13;6:7. doi: 10.1038/s41522-020-0118-1 (PMC7018694; doi:10.1038/s41522-020-0118-1)
Supplement: Supplementary file 1 — Supplementary Information [file 41522_2020_118_MOESM1_ESM.pdf]

Supplementary information

Supplementary Tab. 1. Lists of the plasmids and primers.

**Plasmid**	**Application**	**Reference**
pUG6	deletion cassette (kanMX marker)	[1]
pYM-N1	P_{CUP1} cassette	[2]
pKT127	C-terminal GFP fusion	[3]

**Primer**	**Sequence**	**Purpose**
CYC8-del-forward	AACAACAACAAACAAAACACGACTGGAAAAAAAAAATTAGGAAAACAGCTGA AGCTTCGTACGC	deletion of *CYC8* gene
CYC8-del-reverse	GATTATAAATTAGTAGATTAATTTTTTGAATGCAAACTTTGCATAGGCCACTAGT GGATCTG	deletion of *CYC8* gene
TUP1-del-forward	TGATAAGCAGGGGAAGAAAGAAATCAGCTTTCCATCCAAACCAATCAGCTGAA GCTTCGTACGC	deletion of *TUP1* gene
TUP1-del-reverse	GTTTAGTTAGTTACATTTGTAAAGTGTTCCTTTTGTGTTCTGTTCGCATAGGCCAC TAGTGGATCTG	deletion of *TUP1* gene
CUP1_CYC8 forward	AACAACAAACAAAACACGACTGGAAAAAAAAAATTAGGAAAAATGCGTACGCT GCAGGTCGAC	Insertion of p_{CUP} in front of *CYC8* coding sequence
CUP1_CYC8-reverse	CTGTTGAGCGGGTTGTTCCATTATTGTTTGTTCACCGCCCGGATTCATCGATGAA TTCTCTGTCG	Insertion of p_{CUP} in front of *CYC8* coding sequence
CUP1_TUP1-forward	TAAGCAGGGGAAGAAAGAAATCAGCTTTCCATCCAAACCAATATGCGTACGCTG CAGGTCGAC	Insertion of p_{CUP} in front of *TUP1* coding sequence
CUP1_TUP1-reverse	GCTCATTCAGCTTATTCTGCGTATTCGAAACGCTGGCAGTCATCGATGAATTCTC TGTCG	Insertion of p_{CUP} in front of *TUP1* coding sequence
CYC8-EGFP-forward	AAAATGTAGTAAGGCAAGTGGAAGAAGATGAAAACTACGACGAC GGTGACGGTGCTGGTTTA	C-terminal fusion of *CYC8* with GFP
CYC8-EGFP-reverse	TCTCGTTGATTATAAATTAGTAGATTAATTTTTTGAATGCAAACTTTTTATCGATG AATTCGAGCTCG	C-terminal fusion of *CYC8* with GFP
TUP1-EGFP-forward	TAAAGCAAGGATTTGGAAGTATAAAAAAATAGCGCCAAAT GGTGACGGTGCTGGTTTA	C-terminal fusion of *TUP1* with GFP
TUP1-EGFP-reverse	GTTAGTTACATTTGTAAAGTGTTCCTTTTGTGTTCTGTTC TCGATGAATTCGAGCTCG	C-terminal fusion of *TUP1* with GFP

References

1. Guldener U, Heck S, Fielder T, Beinhauer J, Hegemann JH. A new efficient gene disruption cassette for repeated use in budding yeast. *Nucleic Acids Res*. **24**, 2519-24.(1996)
2. Janke C, Magiera MM, Rathfelder N, Taxis C, Reber S, Maekawa H, et al. A versatile toolbox for PCR-based tagging of yeast genes: new fluorescent proteins, more markers and promoter substitution cassettes. *Yeast (Chichester, England)*. **21**, 947-62.(2004)
3. Sheff MA, Thorn KS. Optimized cassettes for fluorescent protein tagging in *Saccharomyces cerevisiae. Yeast (Chichester, England)*. **21**, 661-70.(2004)

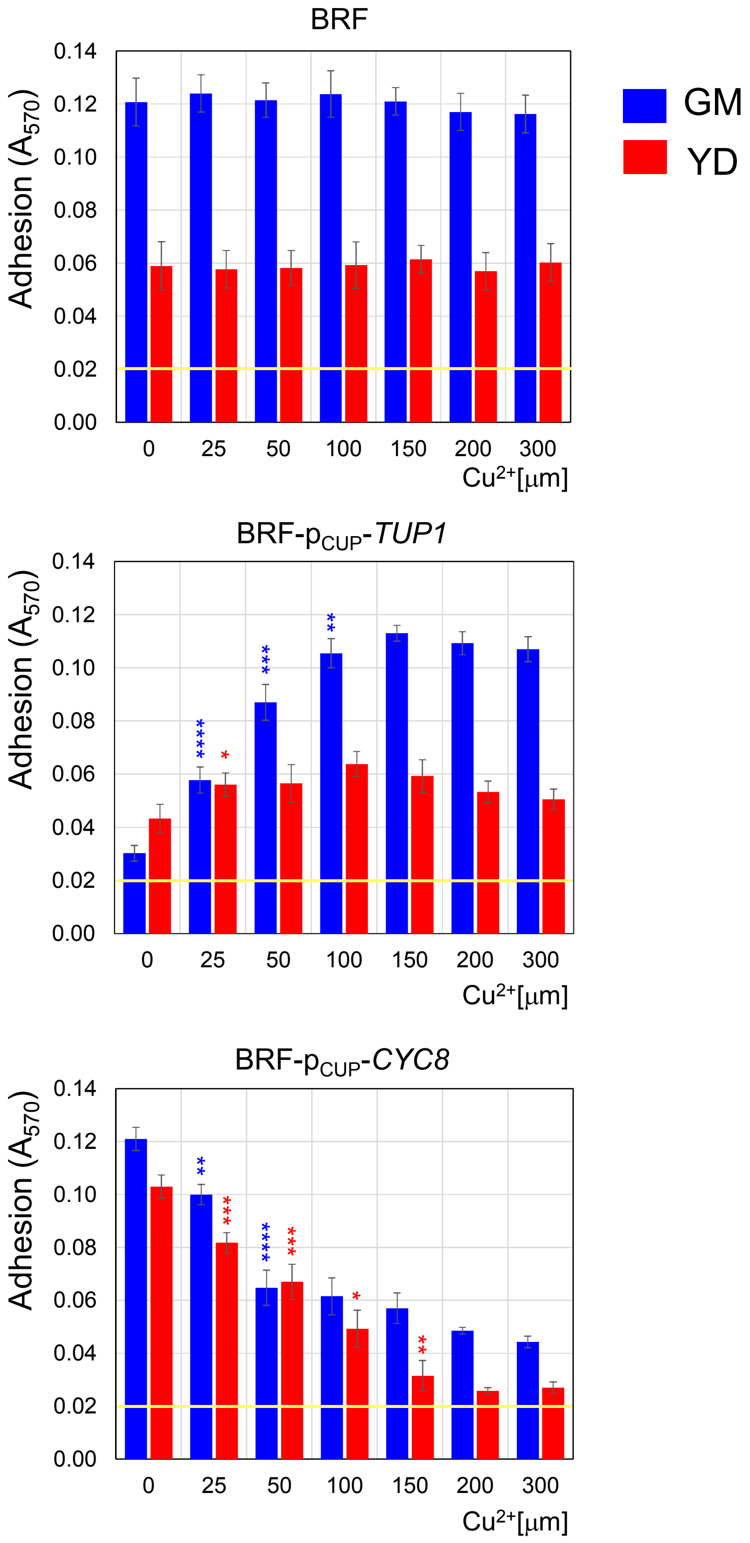

Supplementary Figure 1. **Effect of copper on adhesivity of static BRF, BRF-p$_{CUP}$-*TUP1* and BRF-p$_{CUP}$-*CYC8* cells** in GM and YD. Adhesivity of 24-hrs old static cultures in GM (blue bars) or YD (red bars) containing 0-300 µm Cu^{2+} was analyzed by standard assay. Yellow line indicates BA. Four distinct experimental replicates (*n*=4) were measured for each strain and condition with results expressed as the means and s.d.'s. The statistical significance of the variation between two succeeding galactose concentrations was determined using an unpaired two-tailed t test and GraphPad Prism6 software; **** (p-value < 0.0001), *** (p-value < 0.001), ** (p-value < 0.01) and * (p-value < 0.05).

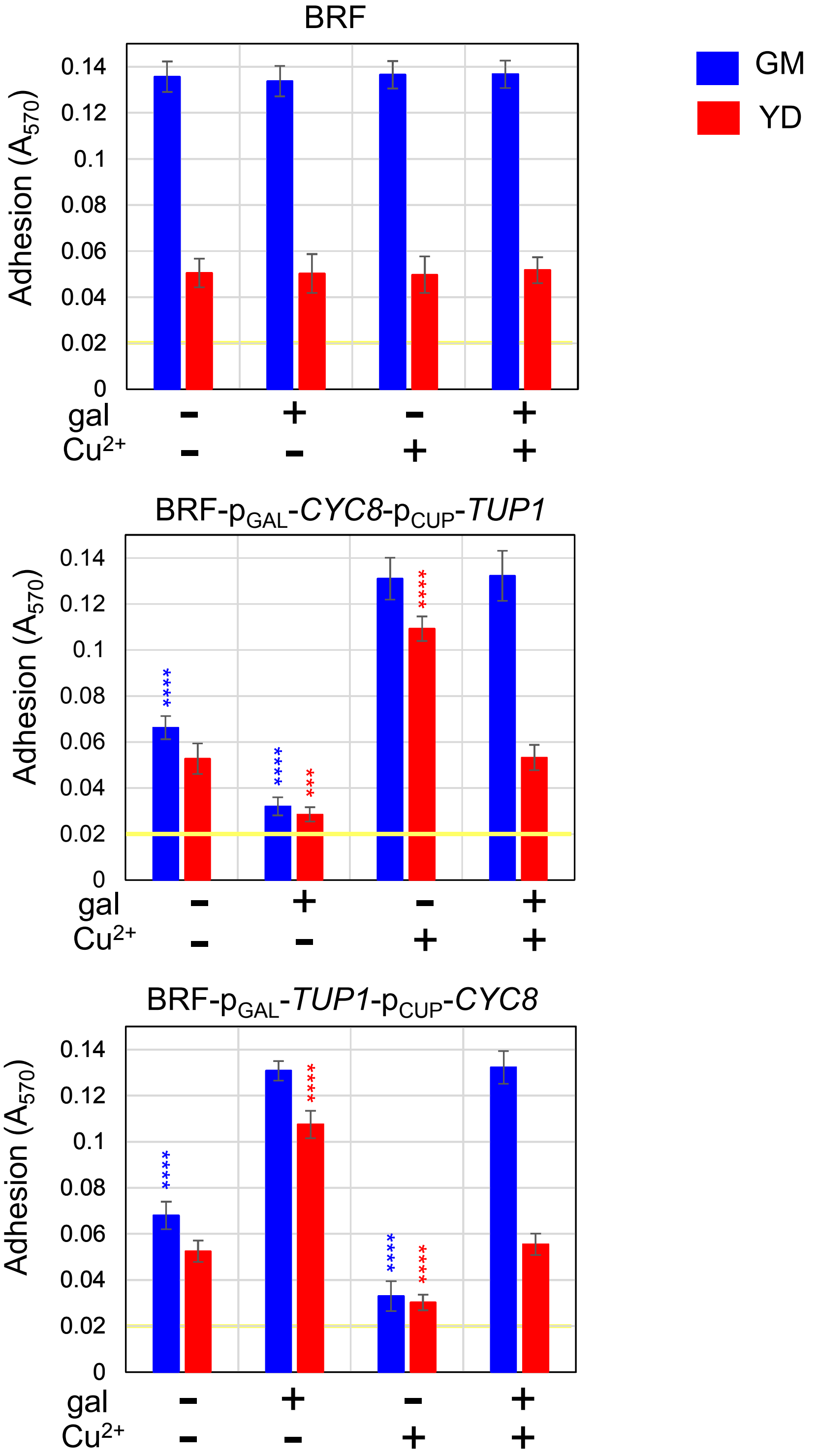

Supplementary Figure 2. Effect of copper and galactose on adhesivity of static BRF, BRF-p_{GAL}-*TUP1*- p_{CUP}-*CYC8* and BRF-p_{GAL}-*CYC8*-p_{CUP}-*TUP1* cells in GM and YD. Adhesivity of 24-hrs old static cultures in GM (blue bars) or YD (red bars) with or without 0.1 % galactose and 300 μM Cu^{2+} as indicated below bar charts was analyzed by standard assay. Yellow line indicates BA. Four distinct experimental replicates (n=4) were measured for each strain and condition with results expressed as the means and s.d.'s. The statistical significance of the variation relative to conditions "+gal +Cu^{2+}" was determined using an unpaired two-tailed t test and GraphPad Prism6 software; **** (p-value < 0.0001) and *** (p-value < 0.001).

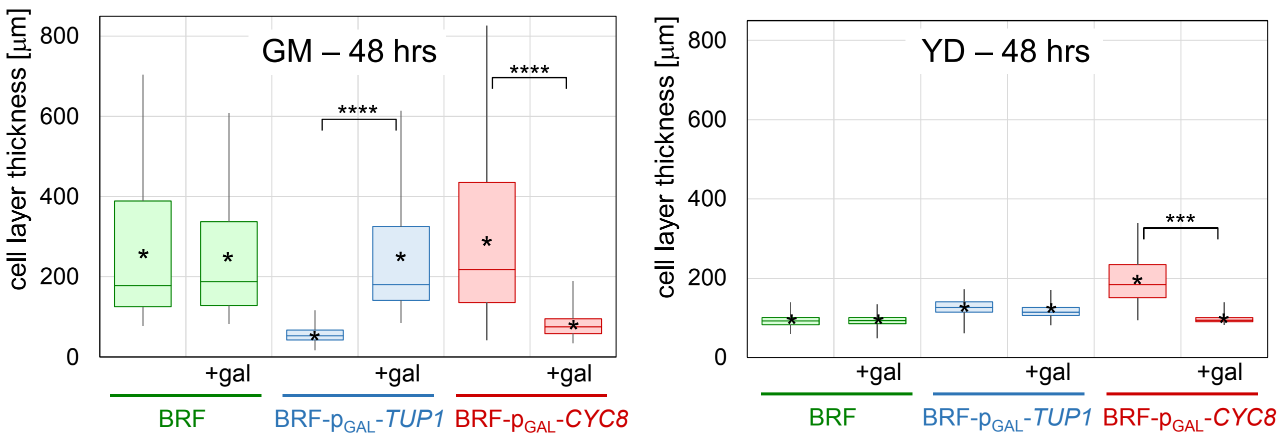

Supplementary Figure 3. Thickness of biofilms and non-adherent cell layers formed by BRF, BRF-p$_{GAL}$-*TUP1* and BRF-p$_{GAL}$-*CYC8* cells. Vertical cross-sections of 48-hrs-old static cultures in GM (left) or YD (right) without galactose or treated with 0.1% galactose (gal) were used for measurement of thickness of biofilm/non-adherent cell layers. The box plots show distribution of thickness measured at different positions (low and high wrinkles) within biofilms and non-adherent cells (box extends from the 25th to 75th percentiles with centre line representing the median and whiskers from Min to Max, asterisk indicates the mean). >200 different positions from 3-5 distinct cross-sections were measured for each plot using ImageJ. The statistical significance of the variation between galactose-treated and untreated samples was determined using an unpaired two-tailed t test and GraphPad Prism6 software; **** (p-value < 0.0001) and *** (p-value < 0.001) .

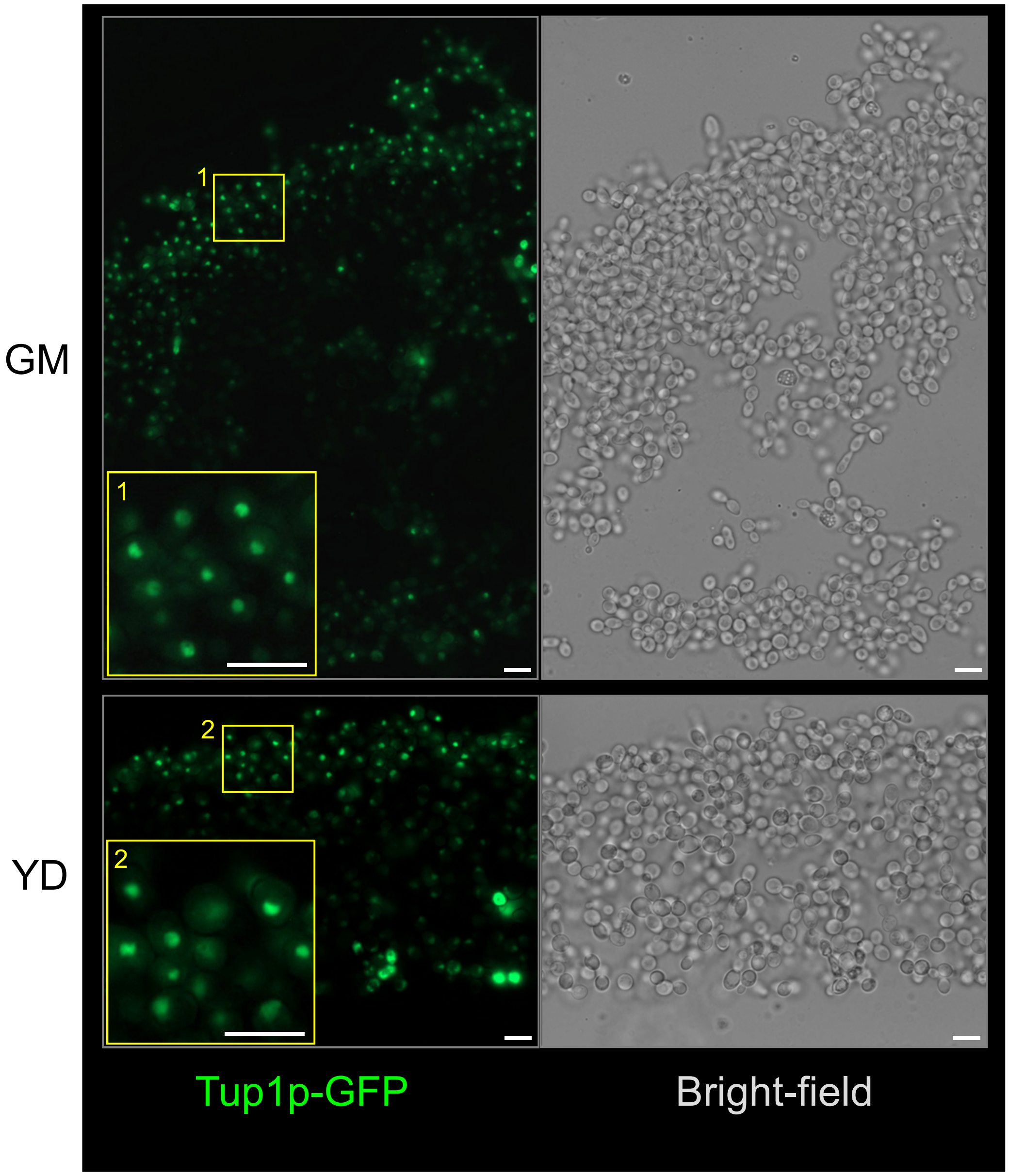

Supplementary Figure 4. Tup1-GFP level in biofilms and non-adherent cell layers. Vertical cross-sections of 24-hrs old static cultures of BRF-Tup1p-GFP strain in GM or YD; Tup1p-GFP fluorescence shown in green, cells are visible in bright-field. Cells in insets "1-2" are shown at higher magnification, indicating intensity of fluorescence of Tup1p-GFP in biofilm/non-adherent cells. Bar, 10 μm. A representative experiment of three (*n*=3) independent experiments is shown.

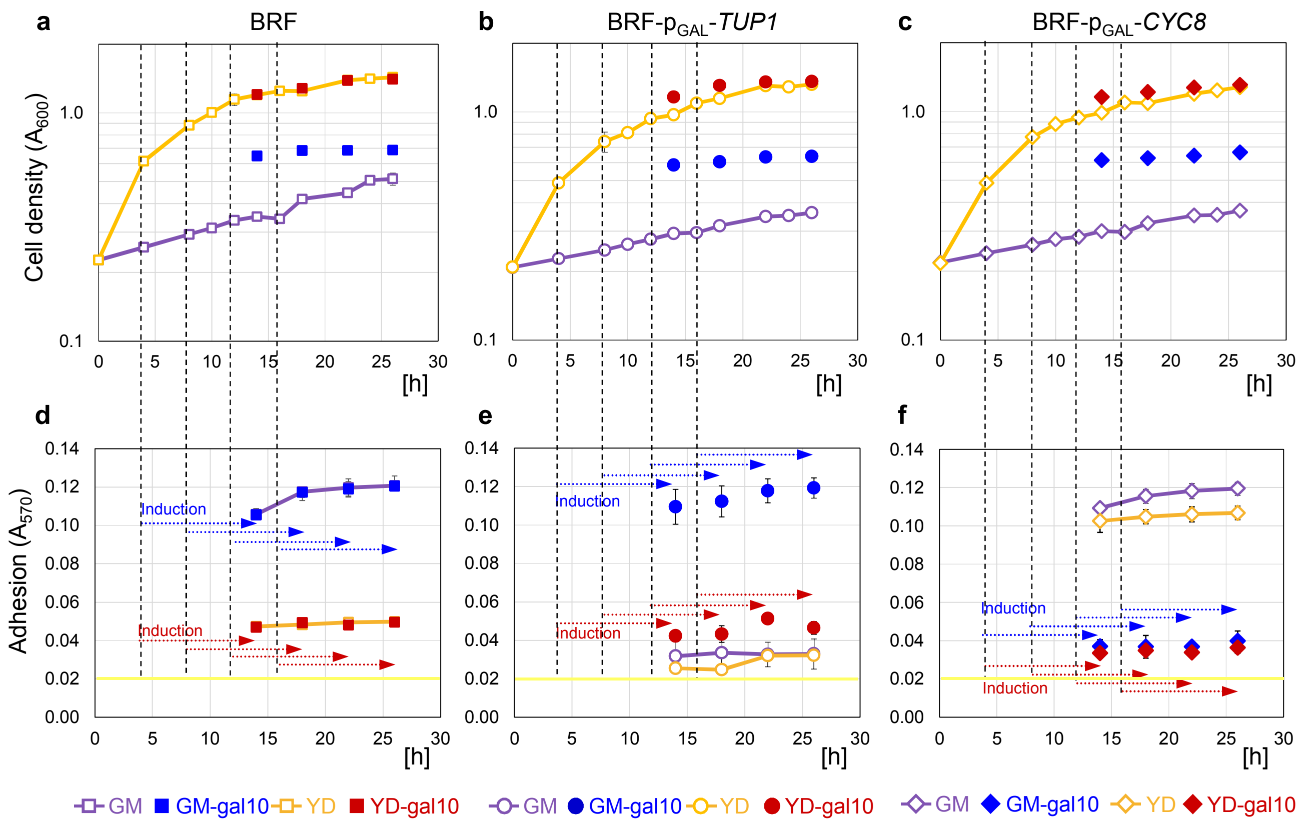

Supplementary Figure 5. Effect of Tup1p or Cyc8p induction on adhesivity of cells in different growth phases of static cultures. a-c. Strain growth curves in GM and YD. gal10, galactose was added 10 hrs before measurement of the biomass (A_{600}). **d-f**, Adhesivity of the strains without induction and after 10 hrs of galactose induction. Arrows (**d-f**) indicate interval of galactose presence in relation to time-points (indicated by dashed lines) in which galactose was added. Yellow line indicates BA. Experiments were conducted in quadruplicate (distinct samples, *n*=4) with results expressed as the means and s.d.'s.

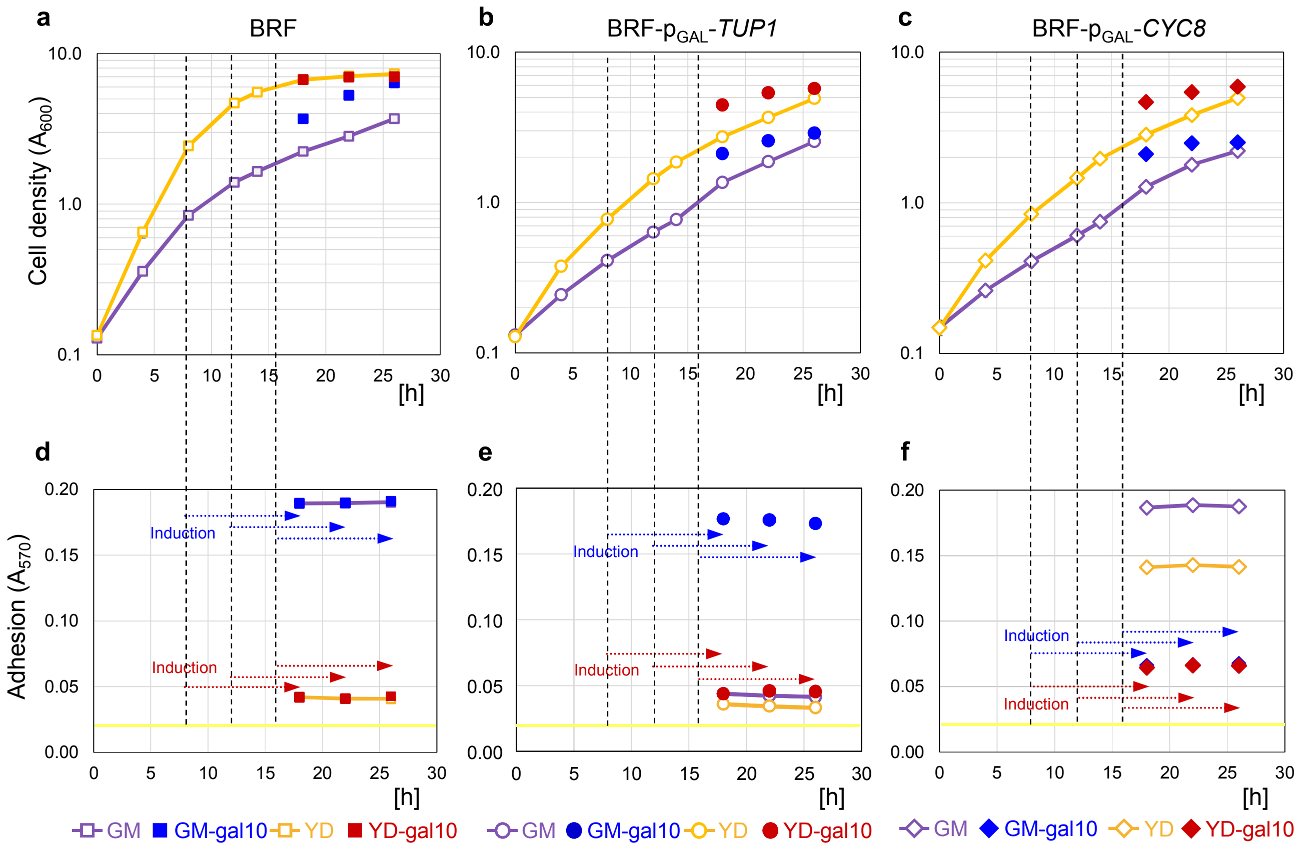

Supplementary Figure 6. Effect of Tup1p or Cyc8p induction on adhesivity of planktonic cells from different growth phases of shaken cultures. a-c. Strain growth curves in GM and YD. gal10, galactose was added 10 hrs before measurement of the biomass (A_{600}). **d-f**, Adhesivity of the strains without induction and after 10 hrs of galactose induction. Arrows (**d-f**) indicate period of galactose treatment in relation to time-points (indicated by dashed lines) in which galactose was added. Yellow line indicates BA. Experiments were conducted in quadruplicate (distinct samples, *n*=4) with results expressed as the means and s.d.'s.
